# Supplementary material for: Eukaryotic-like gephyrin and cognate membrane receptor coordinate corynebacterial cell division and polar elongation
Source: Nat Microbiol. 2023 Sep 7;8(10):1896–910. doi: 10.1038/s41564-023-01473-0 (PMC10522489; doi:10.1038/s41564-023-01473-0)
Supplement: Supplementary file 1 — Supplementary Figs. 1–3, Tables 2, 5 and 6, and References. [file 41564_2023_1473_MOESM1_ESM.pdf]

nature microbiology

Article https://doi.org/10.1038/s41564-023-01473-0

Eukaryotic-like gephyrin and cognate membrane receptor coordinate corynebacterial cell division and polar elongation

In the format provided by the authors and unedited

This PDF file includes:

Figures S1 to S3

Tables S2, S5 and S6

Supplementary References

Note: Tables S1, S3 andS4 are provided separately as an excel file

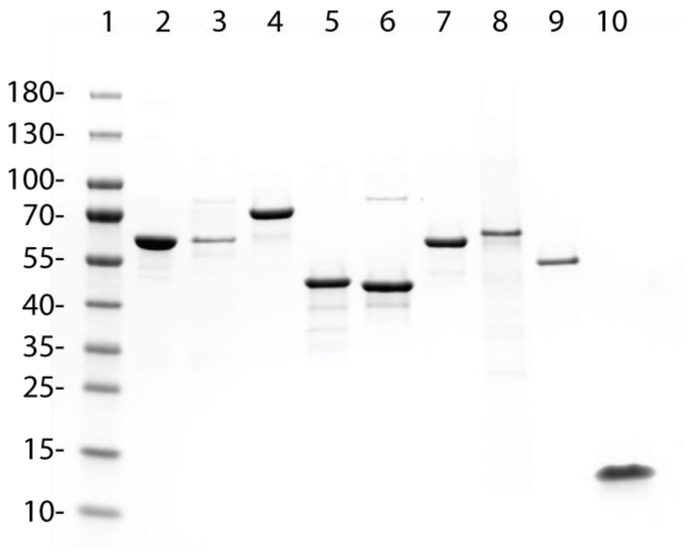

Figure S1. SDS-PAGE on a 4-20% polyacrylamide gel of all purified recombinant proteins used in this work. Lane 1: Molecular weight ladder (kDa); lane 2: His-Sumo-GLP (56.3 kDa); lane 3: His-Sumo-GLP$_{\Delta loop}$ (55.1 kDa); lane 4: His-Sumo-FtsZ (59.2 kDa); lane 5: GLP (44.2 kDa); lane 6: GLP$_{\Delta loop}$ (43 kDa); lane 7: FtsZ (47.2 kDa); lane 8: His-GLPR (40.7 kDa); lane 9: Wag31 (38.7 kDa); lane 10: Wag31$_{1\text{-}61}$ (7.1 kDa). Equivalent migration profiles have been obtained at least three times.

Corresponds to Figure 3c

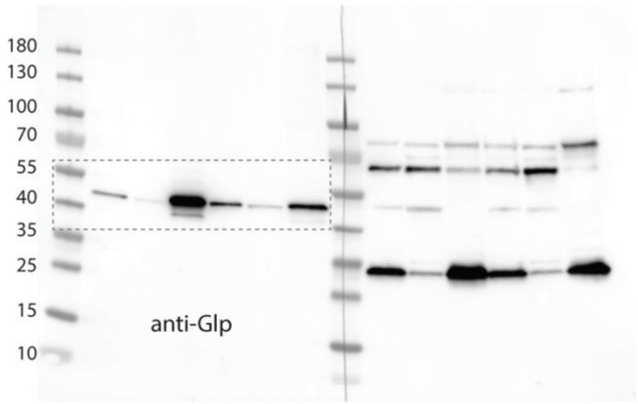

Corresponds to Figure 4g

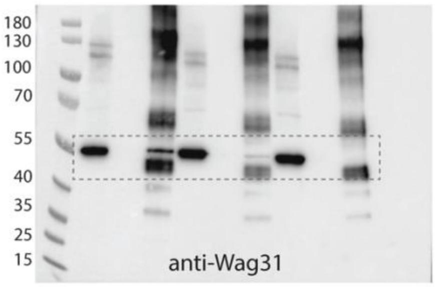

Corresponds to Figure 4g

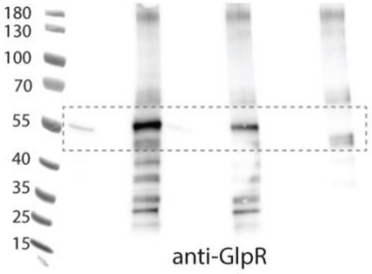

Figure S2. Full uncropped Western Blots of all cropped analyses shown in this work. The boxes correspond to the crops used in the named figures. In Figure 4g, background signals in elution fractions corresponds to the anti-GLPR rabbit antibodies used for the co-IP, which are recognized by the secondary anti-rabbit antibodies used to reveal the Western Blot. Molecular weight markers (kDa) are shown on the left of each blot.

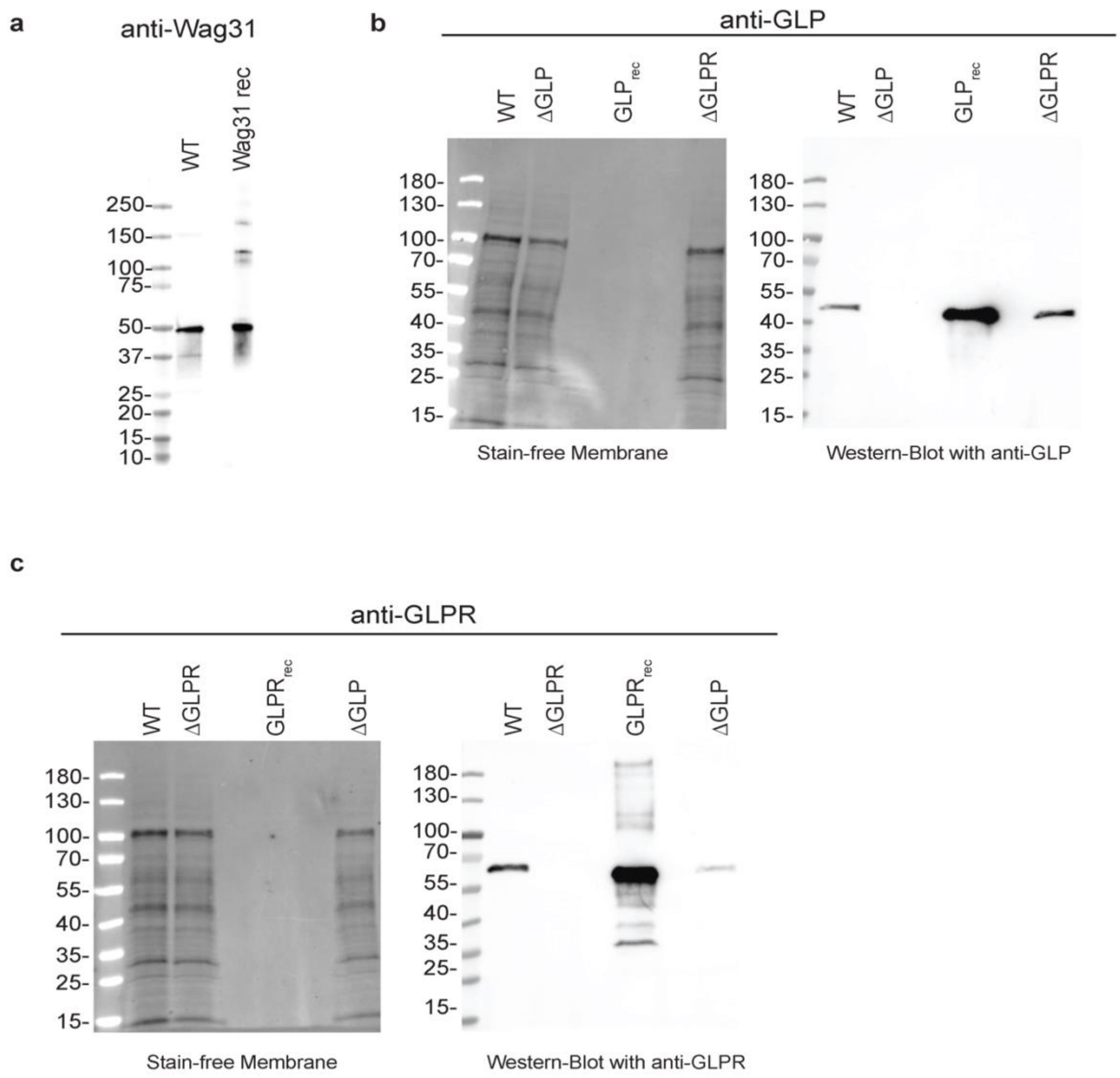

Figure S3. Antibody characterization. (**a**) Western blot of whole cell extracts (120 μg) from *Cglu* strain. Wag31 levels were revealed using the α-DivIVA antibody. 10 ng of recombinant Wag31 were loaded as positive control. (**b**) Stain-free image and Western blot of whole cell extracts (120 μg) from *Cglu, Cglu_Δglp* and *Cglu_Δglpr* strains. Cell lysates were separated on a Criterion TGX Stain-Free SDS gel, transferred onto a Nitrocellulose membrane using the Trans-Blot Turbo Transfer System, and imaged with the ChemiDoc MP imaging system. GLP levels were revealed using the α-GLP antibody. 10 ng of recombinant GLP were loaded as positive control. (**c**) Stain-free image and Western blot of whole cell extracts (120 μg) from *Cglu, Cglu_Δglp* and *Cglu_Δglpr* strains. Cell lysates were separated on a Criterion TGX Stain-Free SDS gel, transferred onto a Nitrocellulose membrane using the Trans-Blot Turbo Transfer System, and imaged with the ChemiDoc MP imaging system. GLPR levels were revealed using the α-GLPR antibody. 10 ng of recombinant GLPR were loaded as positive control. Equivalent Blots have been obtained at least 3 times. Molecular weight markers (kDa) are shown on the left of each blot.

Table S1. Interactomic data. (a) SepF interactome, (b) differential interactome $SepF_{K125E/F131A}$ vs SepF (or derivates), (c) GLP interactome in wild-type *Cglu* background, (d) GLP interactome in *Cglu_Δglpr* background.

See separate Excel Table

Table S2. Crystallographic data collection and refinement statistics.

**Data collection**	**GLP** **Cl_4K_2Pt derivative**	**GLP**	**GLP** **FtsZ-CTD**
Synchrotron Beamline	SOLEIL Proxima 1	SOLEIL Proxima 2A	SOLEIL Proxima 2A
Wavelength (Å)	1.0720	0.9801	0.9801
Space group	$P4_32_12$	$P4_32_12$	$P4_32_12$
Cell dimensions *a*, *b*, *c* (Å)	94.46, 94.46, 230.61	94.44, 94.44, 230.56	95.58, 95.58, 229.52
Resolution (Å)	49.2 – 2.35 (2.41 – 2.35) *	49.2 – 2.14 (2.19 – 2.14)	49.2 – 2.68 (2.81 – 2.68)
*R*pim	0.018 (0.186)	0.023 (0.352)	0.050 (0.296)
I / *s*(*I*)	26.8 (3.9)	21.1 (2.2)	14.6 (2.7)
Completeness (%)	99.7 (96.8)	99.7 (96.2)	99.6 (97.1)
CC(1/2)	1.0 (0.92)	1.0 (0.777)	0.997 (0.800)
Multiplicity	26.8 (26.6)	26.4 (25.0)	9.6 (9.7)
Total observations	1197065	1551838	251139
Unique observations	44612 (3156)	58764 (4332)	30746 (3905)
**Refinement**			
Resolution (Å)		2.14	2.68
No. reflections			
*R*work/ *R*free (%)		0.191 / 0.223	0.190 / 0.239
No. atoms			
Protein		5823	5959
Ligands/ions		-	-
Solvent		457	234
Average B-factors (Å^2)			
Protein		49	42
Ligand/ions		-	-
Solvent		57	50
R.m.s deviations			
Bond lengths (Å)		0.007	0.004
Bond angles (º)		0.876	0.663
Ramachandran favored (%)		98.0	98.6
Ramachandran outliers (%)		0	0.25
**PDB code**		8BVE	8BVF

*Values in parenthesis correspond to the highest resolution shell.

Table S3. Taxonomic sampling of Actinobacteria and protein identifiers of GLP, GLPR and MoeA paralogs. Protein identifiers correspond to the NCBI GenBank database. “NA” indicates that the protein was not identified in the corresponding genome.

See separate Excel Table

Table S4. Taxonomic sampling of Bacteria and protein identifiers of MoeA paralogs. Protein identifiers correspond to the NCBI GenBank database. “NA” indicates that the protein was not identified in the corresponding genome.

See separate Excel Table

Table S5. Plasmids and strains used in this study.

Strains	Characteristics	Reference
E. coli		
DH5α	F- endA1 Φ80dlacZΔM15 Δ(lacZYA-argF)U169 recA1 relA1 hsdR17(rK–mK+) deoR supE44 thi-1 gyrA96 phoA λ–; strain used for general cloning procedures	[1]
CopyCutter EPI400	F- mcrA Δ(mrr-hsdRMS-mcrBC) Φ80dlacZΔM15 ΔlacX74 recA1 endA1 araD139 Δ(ara, leu)7697 galU galK λ- rpsL (StrR) nupG trfA tonA pcnB4 dhfr; strain used for general cloning procedures	[2]
BL21(DE)	F- ompT hsdSB(rB–mB–) gal dcm (DE3); host for protein production	[3]
C. glutamicum		
ATCC 13032	Biotin-auxotrophic wild type	[4]
ΔGLP	*C. glutamicum* ATCC13032 derivative with chromosomic deletion of GLP	This work
ΔGLPR	*C. glutamicum* ATCC13032 derivative with chromosomic deletion of GLPR	This work

**Plasmids for *C. glutamicum* knock out generation**		**Reference**
pK19mobsacB	KanaR; plasmid for allelic exchange in *C. glutamicum*; (pK18 oriVEc, sacB, lacZα)	[5]
pk19-ΔGLP	KanaR; pK19mobsacB derivative for GLP chromosomic deletion	This work
pk19-ΔGLPR	KanaR; pK19mobsacB derivative for GLPR chromosomic deletion	This work

**Plasmids for recombinant protein expression in *E. coli***		**Reference**
pET-SUMO-FtsZ	KanaR; pET derivate for *C. glutamicum* FtsZ recombinant expression containing a N-terminal His-tag followed by a SUMO protease cleavage site	[6]
pET-SUMO-GLP	KanaR; pET derivate for *C. glutamicum* GLP recombinant expression containing a N-terminal His-tag followed by a SUMO protease cleavage site	This work
pET-SUMO-GLP$_{\Delta Loop}$	KanaR; pET derivate for *C. glutamicum* GLP$_{\Delta Loop}$ mutant recombinant expression containing a N-terminal His-tag followed by a SUMO protease cleavage site	This work
pET-SUMO-Wag31	KanaR; pET derivate for *C. glutamicum* Wag31 recombinant expression containing a N-terminal His-tag followed by a SUMO protease cleavage site	This work
pET-His-TEV-Wag31	KanaR; pET derivate for *C. glutamicum* Wag31 recombinant expression containing a N-terminal His-tag followed by a TEV protease cleavage site	This work
pET-His-TEV-Wag31$_{1\text{-}61}$	KanaR; pET derivate for *C. glutamicum* N-teminal DivIVA domain of Wag31 (1-61) recombinant expression containing a N-terminal His-tag followed by a TEV protease cleavage site	This work
pET-SUMO-GLPR$_{IDR1}$	KanaR; pET derivate for the recombinant expression of GLPR IDR1 domain (24-214) containing a N-terminal His-tag followed by a SUMO protease cleavage site.	This work
pET-His-GLPR	AmpR; pET derivate for the recombinant expression of GLPR containing a N-terminal His-tag followed by a TEV protease cleavage site	This work

pET-GLPR-His	KanaR; pET derivate for the recombinant expression of GLPR containing a C-terminal His-tag.	This work

Plasmids for recombinant protein expression in C. glutamicum.		**Reference**
pTGR5	KanaR; *E. coli/C. glutamicum* shuttle vector for regulated gene expression of EGFP under control of tac promoter (Ptac lacI ColE1 oriVEc pGA1 oriVCg)	[7]
pUMS3	KanaR; pTGR5 derivative in which *Ptac* was exchanged by *PgntK* promoter to control the expression of the EGFP protein	[6]
pUMS3-PgntK	KanaR; pUMS3 derivative containing *PgntK* promoter (empty plasmid)	This work
pUMS3-sepF-scarlet	KanaR; pUMS3 derivative for expression of SepF-Scarlet under control of *PgntK* promoter	[6]
pUMS3-sepF$_{K125/F131A}$ -scarlet	KanaR; pUMS3 derivative for expression of SepF$_{K125E\text{-}F131A}$-Scarlet mutant under control of *PgntK* promoter	[6]
pUMS3-Scarlet-I	KanaR; pUMS3 derivative for expression of Scarlet-I fluorescent protein under control of *PgntK* promoter	[6]
pUMS3-GLP	KanaR; pUMS3 derivative for expression of GLP under control of PgntK promoter	This work
pUMS3-mNeon-GLP	KanaR; pUMS3 derivative for expression of mNeonGreen-GLP under control of PgntK promoter	This work
pUMS3-mNeon-GLP$_{\Delta Loop}$	KanaR; pUMS3 derivative for expression of mNeonGreen-GLP$_{\Delta Loop}$ under control of PgntK promoter	This work
pUMS3-GLPR-mNeon	KanaR; pUMS3 derivative for expression of GLPR-mNeonGreen under control of PgntK promoter	This work
pUMS3-GLPR	KanaR; pUMS3 derivative for expression of GLPR under control of PgntK promoter	This work
pUMS3-GLPR$_{\Delta IDR2}$ (1-266)	KanaR; pUMS3 derivative for expression of GLPR with a deletion of the C-terminal IDR2 region under control of PgntK promoter	This work
pUMS3-mNeon-MoeA1	KanaR; pUMS3 derivative for expression of mNeonGreen-MoeA1 under control of *PgntK* promoter	This work
pUMS3-mNeon-MoeA3	KanaR; pUMS3 derivative for expression of mNeonGreen-MoeA3 under control of *PgntK* promoter	This work

Table S6. Oligonucleotide primers used in this study.

Oligonucleotide	Sequence 5' -->3' and properties[a]
**Plasmids for *C. glutamicum* knock out generation**	
**pk19-ΔGLP**	
OligoAS_p68	TGAGCGGATAACAATTTCAC
OligoAS_p69	CAATTCCACACAACATACG
OligoAS_p207	**TGTTGTGTGGAATTG**CTTGCACACTTTGAGCGTTCTTC
OligoAS_p208	**TTACGCATCGAATA**CGGACCTCCTAATCGGAAC
OligoAS_p209	**CCGTA**TTCGATGCGTAATGCACCGTC
OligoAS_p210	**AATTGTTATCCGCTCA**GCAGATTTCTAATGAGATAGCCTTCTGG
**pk19-ΔGLPR**	
OligoAS_p68	TGAGCGGATAACAATTTCAC
OligoAS_p69	CAATTCCACACAACATACG
OligoAS_p216	**TGTTGTGTGGAATTG**ATAAGGAATTCCTCAAGCCCGT
OligoAS_p217	**CTTACCCTGTGG**CTAACCTTCCCGTACGGGTGC
OligoAS_p218	**GTTAG**CCACAGGGTAAGGTTTCGGACTA
OligoAS_p219	**AATTGTTATCCGCTCA**CTGGGAAGTCATACTTCTTGTCCCAC
**Oligonucleotides for KO screening**	
OligoAS_p211	CCTATCGATGAGCACGTGAA
OligoAS_p212	ATGCTTCTCCAGCCTTAGCA
OligoAS_p220	GCACCTATTGGCAGGATTGT
OligoAS_p221	TTCCCACATAACCGAGGAAC

**Plasmids for recombinant protein expression in *E. coli***	
**pET-SUMO-GLP**	
OligoAS_P1	**AGATCCGGCTGCTAAC**AAAGCCCGAAAG
OligoAS_P2	**GAGGCTCACCGCGAA**CAGATTGGTGGC
OligoAS_p169	**CGAACAGATTGGTGGC**GTGCGATCAGTCGAGCAAC
OligoAS_p170	**GTTAGCAGCCGGATCT**CTATCGACCTTGGGCAAGGAA
**pET-SUMO-GLP$_{\Delta Loop}$**	
OligoMM_372	**CATCAGGTCCAGGCTCGGTGTGGAGGCTTTG**GGTGGTGCAACGGGCGCACCATCGCACCTATTGGCA
OligoMM_373	**CAAAGCCTCCACACCGAGCCTGGACCTGATG**AAACCTTTTCGACCCGCCACAGACACAAC

**pET-SUMO-Wag31**	
OligoMM_378	**GCCACCAATCTGTTCGCGGTGA**GCCTC
OligoMM_379	**AGATCCGGCTGCTAACAAAGCC**CGAAAGGAAG
OligoMM_403	**TCACCGCGAACAGATTGGTGGC**ATGCCGTTGACTCCAGCTGATG
OligoMM_404	**GGCTTTGTTAGCAGCCGGATCT**TTACTCACCAGATGGCTTGTTGTTGGTTGGTG
**pET-His-TEV-Wag31$_{1-61}$**	
P1_JP	ATATGTTATTACGCAACCTGCGCCTCCAG
P2_JP	TAATAACATATGGCTAGCATGACTGGTGGAC
**pET-SUMO-GLPR$_{IDR1}$**	
OligoMM_392	**ATGTTCGCCGCCAGCGCTAATTAACCTAGGCTGCTGCCAC**
OligoMM_393	**GCAATGGGCTTTTGGCCTCGGCCACCAATCTGTTCGCGGT**
OligoMM_394	**ACCGCGAACAGATTGGTGGCCGAGGCCAAAAGCCCATTGC**
OligoMM_395	**GTGGCAGCAGCCTAGGTTAATTAGCGCTGGCGGCGAACAT**
**pET-GLPR-His**	
OligoMM_389	**CTTTAAGAAGGAGATATACCATGTCCGGAATCCTTGTGAT**CG
OligoMM_390	**GTGGCAGCAGCCTAGGTTAA**TCAGTGGTGGTGGTGGTGGT
OligoMM_388	**ATCACAAGGATTCCGGACATGGTATATCTCCTTCTTAAAG**TTAAACAA
OligoMM_391	**ACCACCACCACCACCACTGATTAACCTAGGCTGCTGCCAC**
**pET-His-GLPR**	
OligoPB_9	**GGATCCGGCAGCTGGAGCCA**
OligoPB_10	**TCCCTGAAAATACAGGTTTTCCGATCC**
OligoMM_399	**GGATCGGAAAACCTGTATTTTCAGGGA**ATGTCCGGAATCCTTGTGATCGGC
OligoMM_400	**TGGCTCCAGCTGCCGGATCC**TTAGCTCACGCGACGCTGACCCAG

**Plasmids for recombinant protein expression in *C. glutamicum.***	
**pUMS3-mNeon-GLP**	
OligoMM_178	**ATGGTCTTATCCTTTCTTTG**GTGGC
OligoMM_179	**AGCGGCCGCTTAAGGTAC**
OligoMM_286	**CAAAGAAAGGATAAGACCAT**ATGGGTCATCACCATCATCATCACATGGTGTCCAAGGGCGAAG
OligoMM_306	**TGAGCCAGATCCCTTGTACAGTT**CATCCATGCCCATC
OligoMM_305	**AACTGTACAAGGGATCTGGCTCA**ATGCGATCAGTCGAGCA
OligoMM_311	**GTACCTTAAGCGGCCGCT**CTATCGACCTTGGGCAAGGAAGA

**pUMS3-mNeon-GLP$_{\Delta Loop}$**	
OligoMM_372	**CATCAGGTCCAGGCTCGGTGTGGAGGCTTTG**GGTGGTGCAACGGGCGCACCATCGCACCTATTGGCA
OligoMM_373	**CAAAGCCTCCACACCGAGCCTGGACCTGATG**AAACCTTTTCGACCCGCCACAGACACAAC
**pUMS3-GLPR-mNeon**	
OligoMM_178	**ATGGTCTTATCCTTTCTTTGGTGGC**
OligoMM_353	**GGAAGCGGCAGCATGGTGTC**CAAGGGCGAAG
OligoMM_354	**CGCCACCAAAGAAAGGATAAGACCAT**ATGTCCGGAATCCTTGTGATCGGCCTGATTGTGGTGGTGTGGCTTGTTGT
OligoMM_355	**GACACCATGCTGCCGCTTCC**GCTCACGCGACGCTGACCCAGGTGTCGAATGTTTCGACGTGGCTCCTCGTAG
**pUMS3-GLPR**	
OligoMM_374	**GGACACCATGCTGCCGCTTTAGCTCACGCG**ACGCTGACCCAG
OligoMM_375	**CGCGTGAGCTAAAGCGGCAGCATGGTGTCC**AAGGGCGAAGAG
**pUMS3-GLPR$_{\Delta IDR2}$**	
OligoMM_396	**GCTCTTCGCTAACGTCGAATCCG**CAGGCTCCGCCGCTC
OligoMM_397	**CGGATTCGACGTTAGCGAAGAGC**ATTCTCCTCACGAACCTG
**pUMS3-mNeon-MoeA1**	
OligoMM_179	**AGCGGCCGCTTAAGGTAC**
OligoMM_306	**TGAGCCAGATCCCTTGTACA**GTTCATCCATGCCCATC
OligoMM_358	**TGTACAAGGGATCTGGCTCA**ATGTCTCGTTCGCCGGAGCAAC
OligoMM_359	**GTACCTTAAGCGGCCGCT**TTAGTTGAATGGGTAAATCTTAACGATGTCGTTTTCCTC
**pUMS3-mNeon-MoeA3**	
OligoMM_179	**AGCGGCCGCTTAAGGTAC**
OligoMM_306	**TGAGCCAGATCCCTTGTACAGTTC**ATCCATGCCCATC
OligoMM_360	**GAACTGTACAAGGGATCTGGCTCA**ATGGCACAGCAACGCAGCG
OligoMM_361	**GTACCTTAAGCGGCCGCT**TTACATTCTCCCCAGCACAACATCAACCAGAC

**pUMS3-GLP**	
OligoMM_178	**ATGGTCTTATCCTTTCTTTGGTGG**C
OligoMM_179	**AGCGGCCGCTTAAGGTAC**
OligoMM_310	**CCACCAAAGAAAGGATAAGACCAT**ATGCGATCAGTCGAGCAACAGC
OligoMM_311	**GTACCTTAAGCGGCCGCT**CTATCGACCTTGGGCAAGGAAGA

[a]Overlaps for Gibson assembly are written in bold letters.

Supplementary References:

1. Hanahan, D. Studies on transformation of Escherichia coli with plasmids. *J Mol Biol* 166, 557–580 (1983).

2. D, H. *Epicentre Forum* 11, 6 (2004).

3. Studier, F. W. & Moffatt, B. A. Use of bacteriophage T7 RNA polymerase to direct selective high-level expression of cloned genes. *J Mol Biol* 189, 113–130 (1986).

4. Shimono, S. K. S. U. M. Studies on the amino acid fermentation. *J Gen Appl Microbiol* 3, (1957).

5. Schäfer, A. *et al.* Small mobilizable multi-purpose cloning vectors derived from the Escherichia coli plasmids pK18 and pK19: selection of defined deletions in the chromosome of Corynebacterium glutamicum. *Gene* 145, 69–73 (1994).

6. Sogues, A. *et al.* Essential dynamic interdependence of FtsZ and SepF for Z-ring and septum formation in Corynebacterium glutamicum. *Nat Commun* 11, 1641 (2020).

7. Ravasi, P., Peiru, S., Gramajo, H. & Menzella, H. G. Design and testing of a synthetic biology framework for genetic engineering of Corynebacterium glutamicum. *Microb Cell Fact* 11, 147–147 (2012).
