# Supplementary material for: Eukaryotic-like gephyrin and cognate membrane receptor coordinate corynebacterial cell division and polar elongation
Source: Nat Microbiol. 2023 Sep 7;8(10):1896–910. doi: 10.1038/s41564-023-01473-0 (PMC10522489; doi:10.1038/s41564-023-01473-0)
Supplement: Supplementary file 2 — Reporting Summary [file 41564_2023_1473_MOESM2_ESM.pdf]

Corresponding author(s): Rosario Duran
Anne Marie Wehenkel

Last updated by author(s): Aug 03 2023

Reporting Summary

Nature Portfolio wishes to improve the reproducibility of the work that we publish. This form provides structure for consistency and transparency in reporting. For further information on Nature Portfolio policies, see our Editorial Policies and the Editorial Policy Checklist.

Statistics

For all statistical analyses, confirm that the following items are present in the figure legend, table legend, main text, or Methods section.

n/a	Confirmed	
☐	☒	The exact sample size (n) for each experimental group/condition, given as a discrete number and unit of measurement
☐	☒	A statement on whether measurements were taken from distinct samples or whether the same sample was measured repeatedly
☐	☒	The statistical test(s) used AND whether they are one- or two-sided<br>*Only common tests should be described solely by name; describe more complex techniques in the Methods section.*
☐	☒	A description of all covariates tested
☐	☒	A description of any assumptions or corrections, such as tests of normality and adjustment for multiple comparisons
☐	☒	A full description of the statistical parameters including central tendency (e.g. means) or other basic estimates (e.g. regression coefficient) AND variation (e.g. standard deviation) or associated estimates of uncertainty (e.g. confidence intervals)
☐	☒	For null hypothesis testing, the test statistic (e.g. F, t, r) with confidence intervals, effect sizes, degrees of freedom and P value noted<br>*Give P values as exact values whenever suitable.*
☒	☐	For Bayesian analysis, information on the choice of priors and Markov chain Monte Carlo settings
☒	☐	For hierarchical and complex designs, identification of the appropriate level for tests and full reporting of outcomes
☐	☒	Estimates of effect sizes (e.g. Cohen's d, Pearson's r), indicating how they were calculated

Our web collection on statistics for biologists contains articles on many of the points above.

Software and code

Policy information about availability of computer code

Data collection	Microscopy: Zen Blue 2.6 (Zeiss); Soleil synchrotron (site specific data collection software), Biolayer Interferometry (Octet-Red384 V11.1.1.19), Circular dichroism (Aviv 215 software v3.16); Mass spectrometry: nano-HPLC (UltiMate 3000, Thermo) coupled to a hybrid quadrupole-orbitrap mass spectrometer (QExactive Plus, Thermo); Western Blot imaging: ChemiDoc MP Image Lab Touch 3.0.1 (Biorad).
Data analysis	Microscopy (Fiji v2.9.0/1.53t; MicrobeJ v5.130); X-ray crystallography (XDS v 20220110, CCP4 suite v8.0, Phenix v1.20.1, Phaser v2.8.2, Coot v09.8.8, ChimeraX v1.5); Biolayer Interferometry (GraphPad Prism 9); Circular dichroism: Bestsel; Mass spectrometry: Pattern Lab for Proteomics V software; Phylogeny: HMMER package (v3.3.2) jackhammer, mafft (v7.475), MacSyFinder v1.0.5, iTOL v6, BMGE (v1.2).

For manuscripts utilizing custom algorithms or software that are central to the research but not yet described in published literature, software must be made available to editors and reviewers. We strongly encourage code deposition in a community repository (e.g. GitHub). See the Nature Portfolio guidelines for submitting code & software for further information.

Data

Policy information about availability of data

All manuscripts must include a data availability statement. This statement should provide the following information, where applicable:

- Accession codes, unique identifiers, or web links for publicly available datasets
- A description of any restrictions on data availability
- For clinical datasets or third party data, please ensure that the statement adheres to our policy

Atomic coordinates and structure factors have been deposited in the PDB with accession codes 8BVE (Glp) and 8BVF (Glp-FtsZCTD). The mass spectrometry proteomics data have been deposited to the ProteomeXchange Consortium via the PRIDE81 partner repository with the dataset identifier PXD037255 (http://www.ebi.ac.uk/pride/archive/projects/PXD037255). All phylogenetic data used to produce our results is provided as Supporting Data under the following link: https://data.mendeley.com/datasets/265wyk8r3f/draft?a=6d0ecd8f-0adb-4f71-b984-37b7710c3f0a . All materials of this paper can be provided upon reasonable request. Custom scripts will be made available upon request. Source data are provided for all relevant Figures.

Human research participants

Policy information about studies involving human research participants and Sex and Gender in Research.

Reporting on sex and gender	*Not applicable*
Population characteristics	*Nor applicable*
Recruitment	*Not applicable*
Ethics oversight	*Not applicable*

Note that full information on the approval of the study protocol must also be provided in the manuscript.

Field-specific reporting

Please select the one below that is the best fit for your research. If you are not sure, read the appropriate sections before making your selection.

☒ Life sciences ☐ Behavioural & social sciences ☐ Ecological, evolutionary & environmental sciences

For a reference copy of the document with all sections, see nature.com/documents/nr-reporting-summary-flat.pdf

Life sciences study design

All studies must disclose on these points even when the disclosure is negative.

Sample size	For microscopy images no specific sample size was determined, and in general large numbers of cells were analyzed in order to get close to a normal distribution. Other sample sizes were selected based on published research in the field and/or preliminary experimentation. No sample size calculation was performed. Sample sizes for each experiment are described in detail in Figure legends or Methods section.
Data exclusions	For microscopy image analysis: clusters of cells impossible to segment were exluded from analysis, but they were checked manually to verify that no specific phenotypes were excluded. In the analysis corresponding to Figure 4c only cells showing a mean intensity of mNeon fluorescence greater than 35000 were considered, to discard cells that lost the plasmid, due to toxicity of this plasmid. This is stated in the Methods section. No other data were excluded.
Replication	For cellular studies, at least 3 independent replicates were used. Cells were grown in equivalent conditions and harvested at similar optical densities to assure exponential growth. For mass spectrometry at least 3 replicates per condition were used. Details are provided in figure legends and methods section (Statistics and reproducibility).
Randomization	Samples were not randomized as this is not applicable to this study.
Blinding	All samples were collected and analyzed with attributed numbers to avoid any bias in collection or analysis, but further blinding is not necessary for our experiments, as they only involve rational data.

Reporting for specific materials, systems and methods

We require information from authors about some types of materials, experimental systems and methods used in many studies. Here, indicate whether each material, system or method listed is relevant to your study. If you are not sure if a list item applies to your research, read the appropriate section before selecting a response.

Materials & experimental systems

n/a	Involved in the study
☐	☒ Antibodies
☒	☐ Eukaryotic cell lines
☒	☐ Palaeontology and archaeology
☒	☐ Animals and other organisms
☒	☐ Clinical data
☒	☐ Dual use research of concern

Methods

n/a	Involved in the study
☒	☐ ChIP-seq
☒	☐ Flow cytometry
☒	☐ MRI-based neuroimaging

Antibodies

Antibodies used

Anti-GLP (polyclonal, Rabbit, Covalab, custom produced)
Anti-GLPR (polyclonal, Rabbit, Covalab, custom produced)
Anti-DivIVA (polyclonal, Rabbit,Covalab, custom produced)
Anti-mNeonGreen (monoclonal, mouse, ChromoTek, Ref: 32F6)
Anti-Mouse (ECL Mouse IgG, HRP-linked whole Ab sheep, Cytiva, Ref: NA931V)
Anti-Rabbit (ECL Rabbit IgG, HRP-linked whole Ab donkey, Cytiva, Ref: NA934V)
Anti-SepF (polyclonal, Rabbit, Covalab custom produced and described previously (Sogues et al, 2020, Nat Comm)
Anti-mScarlet (polyclonal, Rabbit, Covalab custom produced and described previously (Sogues et al, 2020, Nat Comm)

Validation

Antibodies were validated against the recombinant antigen used for production and when possible in wild-type versus depleted strains of C. glutamicum. This is described in the Methods and Supplementary information of this work for anti-Glp, anti-GlpR and anti-Wag31 and for anti-SepF and anti-mScarlet in Sogues et al, 2020, Nat Comm. All other antibodies are commercially available and validation statements are available on manufacturers website.
